# Maturity Onset Diabetes of the Young—New Approaches for Disease Modelling

**DOI:** 10.3390/ijms22147553

**Published:** 2021-07-14

**Authors:** Dawid Skoczek, Józef Dulak, Neli Kachamakova-Trojanowska

**Affiliations:** 1 Malopolska Centre of Biotechnology, Jagiellonian University, 30-387 Krakow, Poland; dawid.skoczek@student.uj.edu.pl; 2 Department of Medical Biotechnology, Faculty of Biochemistry, Biophysics and Biotechnology, Jagiellonian University, 30-387 Krakow, Poland; jozef.dulak@uj.edu.pl

**Keywords:** MODY, HNF1A, HNF4A, GCK, iPSCs, CRISPR/Cas9

## Abstract

Maturity-onset diabetes of the young (MODY) is a genetically heterogeneous group of monogenic endocrine disorders that is characterised by autosomal dominant inheritance and pancreatic β-cell dysfunction. These patients are commonly misdiagnosed with type 1 or type 2 diabetes, as the clinical symptoms largely overlap. Even though several biomarkers have been tested none of which could be used as single clinical discriminator. The correct diagnosis for individuals with MODY is of utmost importance, as the applied treatment depends on the gene mutation or is subtype-specific. Moreover, in patients with HNF1A-MODY, additional clinical monitoring can be included due to the high incidence of vascular complications observed in these patients. Finally, stratification of MODY patients will enable better and newer treatment options for MODY patients, once the disease pathology for each patient group is better understood. In the current review the clinical characteristics and the known disease-related abnormalities of the most common MODY subtypes are discussed, together with the up-to-date applied diagnostic criteria and treatment options. Additionally, the usage of pluripotent stem cells together with CRISPR/Cas9 gene editing for disease modelling with the possibility to reveal new pathophysiological mechanisms in MODY is discussed.

## 1. Diabetes—Current Classification and Place for Maturity-Onset Diabetes of the Young (MODY)

Diabetes is a chronic, metabolic disease characterised by elevated levels of blood glucose, which uncontrolled over time leads to serious damage of central organs such as the heart, kidneys, eyes and blood vessels. According to World Health Organization (WHO) about 422 million people worldwide have diabetes with 1.6 million deaths directly attributed to diabetes each year. Both the number of cases and the prevalence of diabetes have been steadily increasing over the past few decades. The first WHO classification system for diabetes was published in 1965 and was simple mainly based on the age of the patient: infantile, young, adult and elderly [1].

The next WHO system, published in 1980 and updated five years later, was globally accepted and widely adopted. In these classifications, the two major classes of diabetes were included as insulin dependent diabetes mellitus (IDDM) or type 1 diabetes mellitus (T1DM), and non-insulin dependent diabetes mellitus (NIDDM) or type 2 diabetes mellitus (T2DM). In addition to these, two other classes were added the so-called “other types” plus gestational diabetes mellitus (GDM). In 1999, WHO recommended that the classification of diabetes should encompass not only different aetiological types of diabetes, but also the clinical stages of the disease and reintroduced the terms T1DM and T2DM, as they were omitted in the report from 1985 [2].

**Figure 1:**
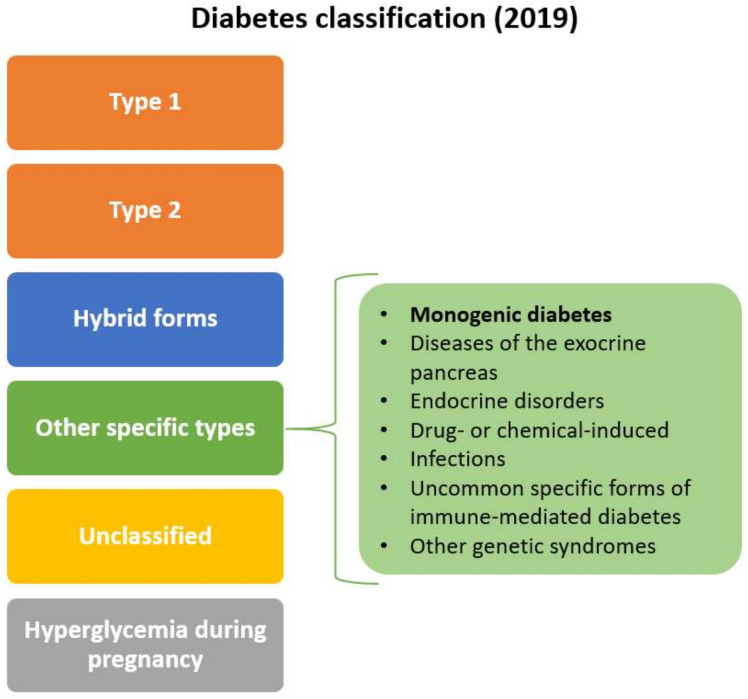
Classification system of diabetes cases (based on WHO, 2019 [5]).

Since the last classification, advances in the knowledge of pathophysiological pathways related to the disease have been made. It became clear that diabetes is a much more heterogeneous disease than the simple classification of T1DM and T2DM. Moreover, many patients have genetic predispositions to both forms of diabetes, which in combination to rapid changes in the environment leads to an increased incidence of the disease worldwide [3]. It was shown that molecular genetics can be used to help in the identification of specific subtypes of monogenic diabetes. Importantly, genetic diagnosis can not only reveal clinical subgroups, but can result in improved treatment outcomes for these patients [4]. All this led to updating the classification system in 2019 [5], that is centred on the β-cell. This enables that appropriate clinical care is delivered according to the international standards set out in the classification system. The β-cell-centric model recognises dysfunctional β-cells as the common denominator of diabetes, which may be caused by either monogenic or polygenic defects in combination to lifestyle factors and environmental changes [6]. In the new classification system, the two major groups (T1DM and T2DM) are included along with so-called “hybrid forms”, “other specific types”, hyperglycaemia during pregnancy and unclassified diabetes. In “other specific types” several subclasses were defined, among them “monogenic diabetes” (Figure 1).

**Figure 2:**
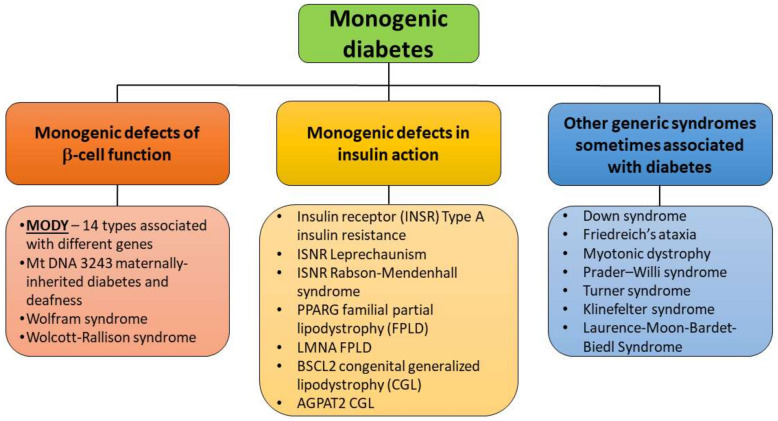
Subclassification of the monogenic diabetes (based on WHO, 2019 [5]).

The monogenic diabetes subclass was additionally divided into monogenic defects of β-cell function, monogenic defects in insulin action and other genetic syndromes associated with diabetes (Figure 2). Clinical manifestation of monogenic defects in β-cell function include maturity-onset diabetes of the young (MODY) and other genetic syndromes where insulin-deficient diabetes is associated with additional clinical features [7,8]. 

## 2. MODY Types

**Figure 3:**
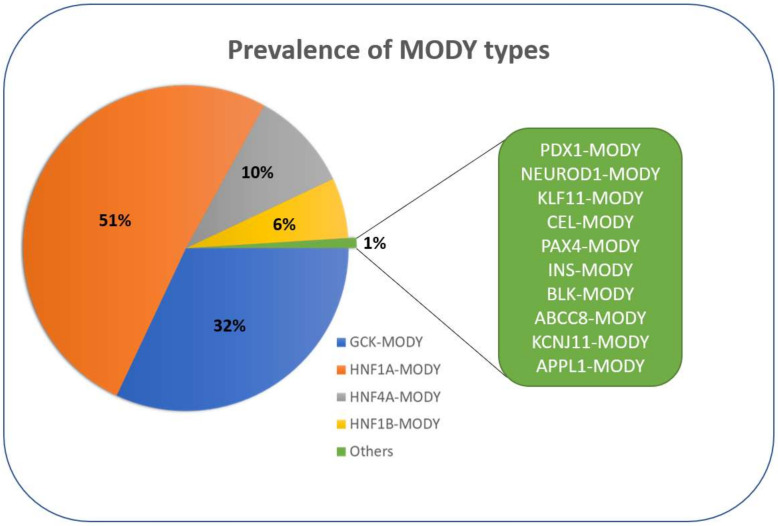
Prevalence of MODY types in some European countries.

MODY is a rare condition, accounting for 1–5% of all diabetes cases [9,10] and 1–6% of paediatric cases of diabetes [11], which means that 1:10,000 adults can be affected [12]. Usually, the definitions of the monogenic subtype come from the gene symbol of the mutated gene followed by the clinical syndrome. The most common forms of MODY are caused by mutations in the glucokinase gene (GCK-MODY) and hepatocyte nuclear factor genes (HNF1A-, HNF1B- and HNF4A-MODY) which together are responsible for around 99% of all MODY cases [13]. HNF1A-MODY is the most frequently found (Figure 3) [14] but the incidence rates vary among different populations. For example, in Japan, around 51.9% cases are caused by unidentified mutations, and among the identified ones *GCK* mutations (22.8%) are the most prevalent [15]. In European countries (UK, Germany, The Netherlands, Norway, Poland), the most common subtype is HNF1A-MODY, followed by GCK-, HNF4A- and HNF1B-MODY [16,17,18,19,20]. Recently, the first MODY cases were reported in patients from Africa, where the predominant subtype was HNF1A-MODY representing 5.9% of the study population [21]. Additionally, mutations in MODY-associated genes were shown to be a significant risk factor for T2DM [22,23,24,25]. However, these big population studies also highlight the high rate of misdiagnosed individuals, which subsequently leads to inappropriate treatment strategies [23,25].

MODY is a monogenic, autosomal dominant form of diabetes with onset before the age of 25, absence of β-cell autoimmunity and impaired β-cell function [10,13]. Currently, 14 MODY subtypes caused by mutations in 14 different genes (Table 1) were described [10,26,27], although mutations in other genes were also described in relation to MODY [28].

## 3. Molecular Pathophysiology of the Most Common MODY Subtypes

All types of MODY are caused by a heterozygous mutation in a specific gene and many of them are considered as haploinsufficiency disorders [29], meaning that one defective allele results in insufficient dose of the gene product leading to failure of the normal cell phenotype. Haploinsufficiency genes are usually expressed during early development and are involved in developmental processes, transcription, cell cycle and nucleic acid metabolism [30,31]. Recently, a homozygous mutation causing MODY has been described [32].

### 3.1. HNF1A MODY

The most common form of MODY is caused by mutations in hepatocyte nuclear factor 1A gene (*HNF1A*). HNF1A is a transcription factor comprised of short N-terminal dimerisation domain, a DNA-binding domain and a C-terminal transactivation domain [33]. It is expressed in liver, kidney, intestine and pancreatic islets [34]. Three functional isoforms of *HNF1A* are described, which are regulated by the same promoter, however they present a different pattern of polyadenylation and are formed by alternative splicing [35]. The longest isoform—*HNF1A*(A)—consists of 10 exons; the shorter is *HNF1A*(B) and the shortest is *HNF1A*(C). These have lengths of 7 and 6 exons, respectively. The expression of these isoforms varies between tissues. In the pancreas, isoform B is the predominant form, while in the liver isoform A is the most abundantly expressed. Isoform C is detected at very low levels in diabetes-associated organs [36].

In the liver, HNF1A is responsible for gluconeogenesis and apolipoprotein synthesis, in the pancreas for the synthesis of the insulin receptor (INSR) and glucose transporter 1 and 2 (GLUT1, GLUT2), whereas in the gut it is supposed to play an important role in controlling terminal functions of the epithelium [37,38,39]. In humans, biallelic deletions of *HNF1A* are not observed because of its impact on development and homeostasis [40,41]. Recently, however, a biallelic mutation of this gene was found to be related to MODY [32] and to cause primary hepatocellular adenoma co-occurring with HNF1A-MODY [41]. Studies with murine *HNF1A^-/-^* model show that knock-out of this gene causes Fanconi syndrome, phenylketonuria, hepatic dysfunction, muscular atrophy, and eventually leads to death in the first weeks after the birth [42,43]. Despite its crucial role in homeostasis, there are studies suggesting a relationship of some *HNF1A* derivatives (especially long noncoding RNA—lncRNA) in progression of cancers such as pancreatic or gastric cancer [44,45].

**Figure 4:**
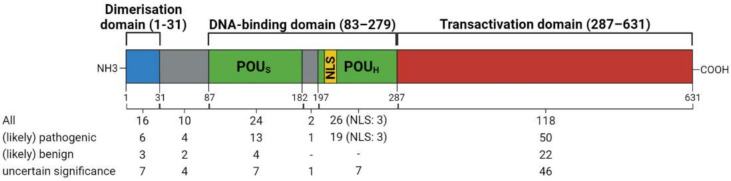
Schematic representation of the HNF1A (isoform A) structure with highlighted functional elements: dimerisation (blue), DNA-binding (green and yellow) and transactivation (red) domains together with the respective amino acids region shown in brackets. The mutations depicted in the figure show 196 of the 198 HNF1A-MODY-related mutations found in ClinVar database and located in the coding sequence of the gene. Two mutations from the database were not included, as they are located in the *HNF1A* promoter region (Supplementary Table S1). The mutations are additionally stratified by clinical significance and the total number of variations in the particular functional domain is presented. Grey boxes—disordered regions; NLS—nuclear localisation signal (range 197–205 amino acids); POU_S_—POU specific domain; POU_H_—POU homeodomain. Figure created with BioRender.com.

The mutations in *HNF1A,* linked to MODY, are heterozygous, scattered throughout the protein coding region, promoter and 5′-UTR and consist of missense, nonsense and frameshift mutations [46,47]. Even though about 375 mutations in the HNF1A gene have been reported to date, 198 of these are associated with HNF1A-MODY or diabetes in general (Supplementary Table S1, based on ClinVar data [48]). It was confirmed that the type of mutation may affect the functionality and stability of the HNF1A protein, and eventually the age of HNF1A-MODY diagnosis [35,49,50,51]. The most common mutations found in the HNF1A gene are missense mutations, which are concentrated in the dimerisation and DNA-binding domains (Figure 4). These truncations are caused by nonsense mutations or, more frequently, by indels resulting in frameshifts and consequently leading to premature stop codons. Depending on the mutations found within *HNF1A,* their effects can be benign, cause MODY, or act as a risk factor for T2DM [52]. Recently, an evaluation of different HNF1A mutations and their effects on clinical parameters were made. The results showed that the best and superior readout that correlated to clinical phenotype was for the transcriptional activity of the gene, therefore it was suggested that new high-throughput functional screens should be developed [53]. Interestingly, a homozygous missense HNF1A (pA251T) variant related to MODY was described recently [32]. The new variant was found to be located in the highly conserved DNA binding domain and the in vitro functional assays demonstrated a modest reduction in transactivation activity and DNA binding of the mutated variant. The homozygous patients presented a similar clinical phenotype to the heterozygous HNF1A-MODY individuals, such as low levels of high sensitivity C-reactive protein (hsCRP) and good responses to sulfonylureas [32].

Recently it was shown that HNF1A may upregulate the transactivation of the anti-apoptotic gene, BCL2 Like 1 (*BCL2L1*). This upregulation was reduced in HNF1A-mutated variants, through inhibiting the transition of G1 to S phase of the cell cycle, thus affecting the cell growth [54]. Additionally, it was suggested that HNF1A-MODY patients can have alterations in their steroid metabolism pathways, which may also explain some of the phenotypic differences, such as normal body weight, in comparison to T2DM individuals [55].

Importantly, HNF1A-MODY patients often develop micro- and macrovascular complications, with retinopathy being one of the most prevalent [56,57]. The incidence of vascular complications is similar to patients with T1DM and T2DM [58]. Moreover, Steele et al. showed that 66% of deaths in HNF1A-MODY carriers may be caused by cardiovascular disease or cerebral vascular events, which is a severe threat to this group of patients. Hyperglycaemia has a negative impact on the cardiovascular system, although the glycaemic index of HNF1A-MODY patients may not be the main reason for this high mortality [57]. The mechanism of cardiovascular impairment in HNF1A-MODY is unclear, but there are suggestions that reduced levels of apolipoprotein M (apoM) in patients with *HNF1A* mutation may be key since the protective role of apoM/S1P axis on the endothelium has been thoroughly described [59,60,61]. Even though the high prevalence of vascular complications in HNF1A-MODY patients was shown in many studies, there are limited data on the underlying mechanisms for such an increased risk for vascular complications. An indirect assessment of the endothelial function in HNF1A-MODY patients showed that these individuals have an increase in intima-media thickness, suggesting abnormalities in endothelial function and presence of early atherosclerotic phenotype [62]. In a more direct approach, an increase in endothelial permeability in response to a proinflammatory cytokine was found in endothelial cells with heterozygous mutation in the HNF1A gene, which were cultured under normal glycaemic conditions, suggesting that endothelial dysfunction was not a due to hyperglycaemia [63]. Other endothelial functions of these cells, such as an increase of intracellular adhesion molecule 1 (ICAM-1) after TNFα stimulation and angiogenic response, were not affected by the HNF1A mutation [63].

### 3.2. HNF4A-MODY

Hepatocyte nuclear factor (HNF) 4A is a transcription factor that belongs to the steroid/thyroid hormone receptor superfamily and controls the expression of many genes associated with critical metabolic pathways [64,65,66]. It contains two zing finger domains, one DNA binding region and requires homodimerisation to bind its recognition DNA site in the nucleus. HNF4A is expressed mainly in the liver, but it can be also found in kidneys, pancreatic islets, and intestine [67,68]. It is engaged in many processes such as glucose entry, lipid and drug metabolism pathways and it may be involved in amino acid metabolism [64,67,69].

HNF4A-MODY was identified back in 1978 in a family known as the RW family and at that time was the first well-identified MODY type [70,71]. Heterozygous mutations in *HNF4A* are relatively rare and constitute 5–10% of all MODY cases. Till 2013, 103 different mutations were reported in 173 families [72], whereas currently there are around 180 mutations in *HNF4A* associated with MODY (ClinVar [48]). Mutations in HNF4A were shown to cause increased insulin production in the human foetus, causing faster growth, higher body weight and even macrosomia. The hyperinsulinaemic hypoglycaemia can be detected in the early life of HNF4A-MODY carriers, leading to MODY manifestation later on [73,74,75]. This is thought to reflect a switch later in life to defective insulin secretion, although a prolonged hyperinsulinaemic phase in adulthood was described as well [74]. Therefore, HNF4A-MODY induced transient neonatal hyperglycaemia may precede the later diabetes onset [76]. However, the precise mechanism and timing of this transition remains unclear.

Several types of mutations (missense and nonsense) in *HNF4A* have been linked to altered insulin secretion [77], where the K_ATP_ channel in murine β-cells may play a role in the dysfunction [78]. In β-cells, HNF4A mutations cause dysfunction or lipid profile disruption, which is probably mediated through genes involved in glucose metabolism and biosynthesis [64]. These include GLUT2, aldolase B, liver pyruvate kinase, insulin, mitochondrial uncoupling protein-2 and glyceraldehyde-3-phosphate dehydrogenase [79,80]. However, most of the HNF4A mutations do not demonstrate a dominant-negative effect on the gene expression profiles, and it is still not clear how moderate decreases in HNF4A activity cause disease in MODY patients [81].

In addition, some studies suggest that single nucleotide polymorphism in the HNF4A gene may predispose to T2DM, at least in some populations [82,83,84,85].

### 3.3. GCK-MODY

The glucokinase (*GCK*) gene has a crucial role as a glucose sensor and integrates glucose metabolism with insulin secretion in pancreatic β-cells [86]. It codes for an enzyme responsible for catalysis of the first glycolysis reaction. GCK is highly expressed in hepatocytes, pancreatic β-cells and in the brain. Mutations in this gene are responsible for various glucose regulation disorders, with GCK-MODY cases among them. The most frequent mutations are heterozygous inactivating mutations leading to alterations in the kinetic parameters of this enzyme [87]. Patients with GCK-MODY show a defect in glucose sensing, therefore the glucose homeostasis is maintained at a higher set point. This results in mild and asymptomatic fasting hyperglycaemia, which is present from birth [88]. The median age of hyperglycaemia diagnosis is around 24 years of age [87]. GCK-MODY patients are characterised by increased fasting glucose levels, but the majority do not require any pharmacotherapy. The exception are pregnant women, who should be treated with insulin to reduce the risk for diabetic foetopathy in the child, which is not a carrier of the mutation [89]. The foetal genotype is not usually known, but an assumption can be made based on serial ultrasound measurements. In case there is no evidence for accelerated growth, the foetus is assumed to have inherited the *GCK* mutation, and in such circumstance the maternal hyperglycaemia is not treated [90].

Even though patients with GCK-MODY have long-lasting hyperglycaemia, these individuals show low prevalence of micro- and macrovascular complications [91].

### 3.4. HNF1B-MODY

Hepatocyte nuclear factor 1B (HNF1B) is a transcription factor expressed in many organs, predominantly in the liver, intestine, pancreas and kidney. Importantly, the expression of HNF1B also occurs in the pre-pancreatic foregut endoderm and in pancreatic multipotent progenitor cells (MPCs), therefore it has an important role in pancreatic differentiation. Lineage tracing studies have revealed that embryonic cells expressing HNF1B are precursors of acinar, duct and endocrine lineages in the pancreas [92]. Using murine models, the important role of HNF1B in the proliferation and survival of the MPCs was confirmed [93]. Heterozygous mutations in the human *HNF1B* are associated with MODY, which is characterised by early onset diabetes, pancreas hypoplasia and multicystic kidney dysplasia, but also with kidney diseases and multi-organ disorders [94,95,96]. Based on murine studies, it was suggested that MODY might occur not only as a consequence of β-cell dysfunction, but also as a consequence of developmental defects, leading to diabetes later in life [93].

Recently, several novel mutations in *HNF1B* and their relation to MODY were reported [97,98,99]. In a human-related study, it was shown that a heterozygous mutation in *HNF1B* caused decreased transcriptional activity, reduced DNA binding and decreased expression of the GLUT2 gene. Based on these results, the authors conclude that the impaired insulin secretion in this family is related to the reduced GLUT-2 expression in β-cells rather than decreased insulin expression [100]. In patients with HNF1B-MODY presence of cystic kidneys, pancreatic abnormalities and elevated liver enzymes are common and were used as predictors of HNF1B mutations [101]. Similarly, the presence of renal/pancreatic abnormalities in young patients with diabetes are suggestive for genetic testing for HNF1B-MODY [102,103].

Diabetes complications and cardiovascular risk factors are highly prevalent in individuals with HNF1B-MODY. In these patients, both diabetic retinopathy and neuropathy were found; however, the major complications were related to kidneys, as chronic kidney disease was reported for about 44% of the studied HNF1B-MODY patients [104].

### 3.5. Other MODY Types

The MODY subtypes described above account for more than 99% of all MODY cases. With the increase in next-generation sequencing (NGS) capabilities, other rarer MODY cases have been reported [105,106]. The current knowledge for possible molecular pathophysiology in rarer forms of MODY were extensively summarised elsewhere [107,108,109].

## 4. Diagnosis and Current Treatment Options

### 4.1. Diagnosis of MODY Patients

The diagnosis of MODY is relatively difficult, as many of the symptoms are highly similar to T1DM and T2DM, which usually leads to misdiagnosis. On the other hand, there is a big clinical variability between the different MODY subtypes, which makes proper diagnosis extremely hard without genetic testing. It should be noted that mutations in some MODY-associated genes (*HNF1A, ABCC8, HNF4A, GCK, KCNJ11*) can cause congenital hyperinsulinism and hypoglycaemia in infants and children preceding the later diabetes onset [110,111,112]. Moreover, even though a family history of diabetes is highly suggestive for MODY, some mutations in MODY-associated genes can occur in high frequencies also de novo, showing the importance of genetic testing in individuals without a family history of diabetes [113].

Even though patients with MODY were found to differ from T1DM patients in several clinical predictors, such as C-peptide concentration [114], hsCRP [115,116,117], lipid levels, polyuria or age at diagnosis, still around 38% of the MODY patients are misdiagnosed with T1DM or T2DM [27,87,118,119,120]. In 2013 Steele et al. reported that age-related glycated haemoglobin (HbA1c) reference ranges can be used as diagnostic criteria for GCK-MODY discriminator [121]. Later on, a combination of markers were tested for a discrimination of individuals with common types of diabetes and MODY [58]. It was shown that hsCRP and 1,5-anhydroglucitol (1,5-AG) could only be used to distinguish HNF1A-MODY from other MODY subtypes, but not from T1DM or T2DM [58]. In two recent population studies, it was suggested that screening for monogenic biomarkers (endogenous insulin secretion, the ratio of urinary C-peptide and creatinine (UCPCR), islet autoantibodies), is an effective, cheap and easily implemented approach for systematic screening of young patients [122,123]. The diagnostic process included three stages, where positive UCPCR patients were further tested for islet autoantibodies, and if negative were then selected for genetic testing [123]. However, the clinical reliability of UCPCR for distinguishing MODY patients from T2DM, was not confirmed [124]. Therefore, the improved diagnosis will require the application of several biomarkers together, but ultimately the genetic test is the best form of diagnosis of MODY patients.

To complicate matters further, some MODY types have similar pathology, which can also lead to misdiagnosis. Some symptoms of HNF4A-MODY, such as transient neonatal hyperinsulinemic hypoglycaemia, progressive insulin secretory defect or microvascular complications, are very similar to symptoms observed in patients with HNF1A-MODY [119,125,126]. Due to these very similar phenotypes, and because HNF1A-MODY is more prevalent, some HNF4A-MODY cases may be incorrectly recognised as HNF1A-MODY. One study suggests that up to 29% of HNF4A-MODY cases may be incorrectly credited to HNF1A-MODY and sequencing of *HNF4A* is proposed when there is no mutation in *HNF1A* [127]. Recently, it was shown that single nucleotide polymorphism located in the HNF1A gene promoter can affect the binding of HNF4A and subsequently regulate HNF1A gene expression [128].

Additionally, HNF4A-MODY can have mild and atypical clinical presentation, again hampering correct diagnosis [129]. There are also individual features of HNF4A-MODY. One of the most described phenotypes is a lower concentration of HDL-cholesterol corresponding to reduced levels of apoA2, B and C3 [64,127,130]. However, the reduced level of triglycerides and apoC3 is questionable [127]. On the other hand, in a study which screened diabetic patients under the age of 20, it was showed that 10% of all HNF4A-MODY cases present dyslipidaemia [118]. The clinical presentation of HNF1A- and HNF4A-MODY could be similar, because these two genes can regulate each other [131].

Currently it is suggested that genetic tests for MODY should be performed when paediatric diabetes is diagnosed, together with modest hyperglycaemia and absence of all four islet autoantibodies (antibodies against GAD, insulinoma antigen-2, zinc transporter 8 and insulin), but there is no standardised diagnostic algorithm [12,132,133]. At present, genetic tests for MODY are conducted with NGS methodology due to lower costs and increased diagnostic accuracy [134].

### 4.2. Current Treatment Options

As mentioned above, the majority of MODY patients are initially misdiagnosed with T1DM or T2DM and because of that are inappropriately treated [16,135,136]. The diagnosis of MODY has significant implications for diabetes management. For example, in patients with GCK-MODY, due to a higher basal glucose level the glucose-lowering therapies are ineffective, therefore the treatment of these patients is not recommended [88]. On the other hand, patients with HNF1A- or HNF4A-MODY are responsive to low-dose sulfonylureas, due to the increased pancreatic insulin secretion [126,137,138,139].

The importance of the correct diagnosis was highlighted in a UK population study [16]. Following genetic diagnosis for MODY, patients with GCK-MODY, who were inappropriately treated at the time of diagnosis, were able to stop this treatment without any effect on HbA1c levels. However, only 58% of HNF1A/HNF4A-MODY patients, who were also treated inappropriately in the past, could change the treatment to sulfonylurea/diet alone. In those individuals with longer duration of diabetes, it was recommended that sulfonylurea should be used together with the previous treatment started at the time of misdiagnosis [16]. The lack of response to sulfonylurea treatment in HNF1A-MODY, when diabetes duration is long-standing and mistreated, was recently confirmed in a pair of siblings with a novel HNF1A variant [140].

Even though HNF1A- and HNF4A-MODY patients are responsive to sulfonylurea, this therapy has a significant risk of hypoglycaemia. Therefore, a combination with other glucose-lowering agents such as dipeptidyl peptidase-4 inhibitor [141,142] or monotherapy with glucagon-like peptide (GLP-1) receptor agonists were also tested [143,144]. Recently, the usage of incretin hormone glucose-dependent insulinotropic peptide (GIP) and GLP-1 were tested in patients with HNF1A-MODY. These therapeutics were used together with sulfonylurea, and the results suggest that such combinations could be beneficial for HNF1A-MODY patients due to the increase of the glucose-stimulated insulin secretion [139].

To summarise, the proper recognition and MODY diagnosis is of utmost importance for the proper treatment of diabetes. The patients should be followed to determine the efficacy of the treatment and to monitor the possible vascular complications in HNF1A-and HNF4A-MODY subtypes. The most recent therapeutic approaches for MODY patients were summarised recently by Broome et al. [145].

## 5. Pluripotent Stem Cells for MODY Disease Modelling and Drug Research

**Figure 5:**
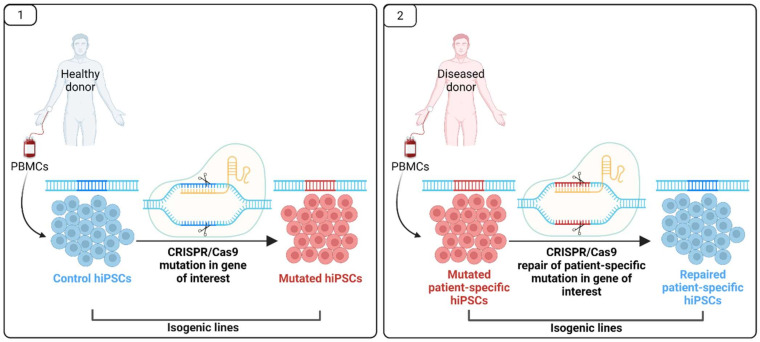
Approaches for generation of human disease-relevant isogenic lines: (**1**) starting from healthy or (**2**) diseased individual. Figure created with BioRender.com.

In most MODY subtypes, the exact pathological mechanisms of disease progression are still unknown. This is due to the inaccessibility of human pancreatic tissue and the fact that rodent models do not recapitulate the MODY disease phenotype [146,147,148], with the exception of HNF1B-MODY [149]. Therefore, currently human-induced pluripotent stem cells (hiPSCs)-based disease modelling tools are being developed aiming to resolve some of the pathological mechanisms of MODY, and possibly reveal new therapeutic strategies for the patients. However, for the development of disease models, which may be used as drug-target platforms, hiPSCs must effectively differentiate and fully recapitulate the hallmarks of diseased cells and tissues. Moreover, the differentiated cells should represent homogeneous and if possible mature cell population, thus reducing the inter- and intra-experimental variation [150,151]. Additionally, to ensure valid disease modelling, the proper control lines should be selected. For monogenic diseases, as MODY, the most stringent controls are the so-called isogenic cell lines (Figure 5). These can be obtained through CRISPR/Cas9 gene editing in two ways: (1) using hiPSCs from healthy individual and introducing the mutation in the gene of interest [63], or (2) through repair of the gene-specific mutation in iPSCs derived from the diseased individual [152]. These two approaches have advantages and drawbacks. The advantages of the first approach are the relatively fast delivery of such mutated hiPSCs lines and that the observed effects will be mutation-specific, excluding the contribution of the genetic background. In the second approach, more patient-specific effects could be identified; however, such repaired hiPSCs line are hard to obtain and are dependent on the patient-specific genetic background. However, both approaches give the opportunity to identify disease-relevant phenotypes through mechanistic studies.

Up to date several hiPSC lines from different MODY types were developed [153,154,155,156]. Even though MODY is a monogenic disease, meaning that this disease is easy to model, there are limited studies using MODY patient-derived cells as disease modelling tools. One such study used patient-derived hiPSCs with a mutation in the HNF1B gene and, as a control, the authors used a non-family control hiPSCs line, and also hiPSCs from a non-diseased family member [157]. The authors were able to show that the mutant HNF1B gene expression was responsible for the compensatory increase in PDX1 gene expression in differentiated pancreatic progenitors [157]. In a different study, patient-derived iPSCs with *HNF4A* mutation upon differentiation toward insulin producing β-cells showed that mutation in this gene is not affecting the expression of insulin genes, nor the development of insulin-producing cells in vitro [158]. The results were obtained with patient-derived hiPSCs from several family members, and one of them, without mutation in HNF4A gene, was used as a non-diseased control [158]. A similar approach was used in a more recent study; however, the iPSCs were differentiated toward hepatopancreatic progenitors (HPPs), and alterations in hepatic and pancreatic β-cell gene signatures were found [156]. Moreover, immunofluorescent analysis showed that HNF4A protein is predominantly localised in the cytoplasm, and this mislocalisation could further account for the loss of function of HNF4A as a transcription factor [156].

There are only a few studies that used isogenic pluripotent stem cell lines to model MODY [63,152,159,160]. In all studies, CRISPR/Cas9 was used to introduce or repair the mutation in the gene of interest; however, the pluripotent stem cell source was different. In two of the studies, embryonic stem cells (ESCs) were used to introduce the mutation in the gene of interest and subsequently they were differentiated toward pancreatic β-like cells [159,160]. Cardenas-Diaz et al. found that loss of HNF1A led to an increase in alpha cell gene expression markers such as glucagon, and decreased *PAX4* expression, which is crucial in regulating the development of β-cells. Moreover, these cells had impaired insulin secretion together with defects in glycolysis and mitochondrial respiration [159]. In the other study with ESCs, Zeng et al. introduced biallelic mutation in KCNJ11 gene and found that there was impaired insulin secretion together with defective glucose homeostasis. However, these cells did not show increased sensitivity to gluco- or lipotoxicity, checked with 35 mM D-glucose or 1 mM palmitate treatment [160].

**Figure 6:**
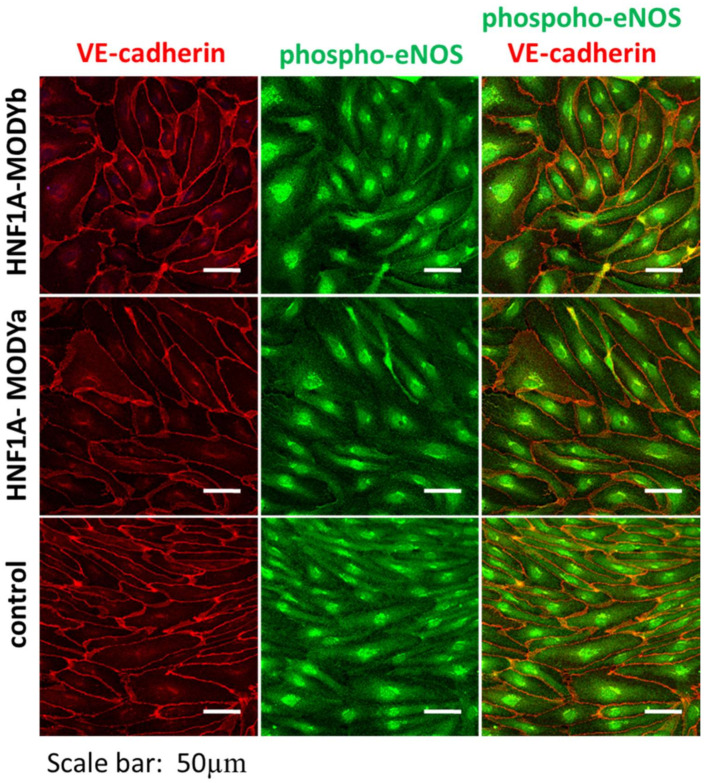
hiPSC-derived endothelial cells differentiated as described in [63]. Cells were derived from healthy (control) and two HNF1A-MODY individuals (HNF1A-MODYa and HNF1A-MODYb). No difference in the expression of VE-cadherin (red) or phosphorylated form of endothelial nitric oxide synthase (phospho-eNOS, green) could be observed.

In our study, hiPSCs derived from healthy donors were used to introduce a mutation in the HNF1A gene [63]. As HNF1A-MODY patients frequently develop vascular complications, the study aimed to check whether a mutation in the HNF1A gene could affect endothelial cell function. The differentiation of hiPSCs toward endothelial cells (hiPSC-ECs) was not affected by introducing *HNF1A* mutations. As shown in Figure 6, hiPSC-ECs derived from healthy or HNF1A-MODY individuals have similar expression of endothelial markers that are crucial for their functioning. Moreover, the majority of the endothelial functional parameters, such aspro-angiogenic responses, were not changed; however, an increase in the vascular permeability after stimulation with a pro-inflammatory cytokine was observed in hiPSC-ECs with the *HNF1A* mutation. These results could suggest that patients with HNF1A-MODY have increased susceptibility to the development of vascular complications [63]. The only study in which repaired patient-specific lines were used was performed by Balboa et al. [152]. They used hiPSCs from Finnish people with a mutation in the INS gene and differentiated them together with the respective isogenic control lines to β-like cells. The single-cell RNA sequencing showed increased expression of endoplasmic reticulum (ER) stress-associated genes, together with reduced proliferation. In vivo, the mutated cells had lower insulin secretion and increased levels of ER-stress markers [152].

All studies using pluripotent stem cells as disease modelling tools are summarised in Table 2.

While in vitro analysis of single cell subtypes is an excellent resource to study linage-specific disease mechanisms, currently more attention is given to developing more complex disease modelling tools. Organoid cultures require 3D growth, and during this process stem cells aggregate and differentiate in response to biophysical cues, resulting in complex structures that mimic the mature organ [161,162,163]. The advances in this field were recently reviewed by Sharma et al. [164]. In diabetes research, the current trend is moving toward developing islet organoids, which can provide large amounts of functional islets and therefore be used in underpinning disease mechanisms in vitro [165,166].

## 6. Final Remarks

Pathophysiological mechanisms of MODY are still not well understood. However, the usage of genomic-based approaches gives an excellent opportunity for gaining knowledge in the biology that results from the specific gene mutations in MODY, and can likely help to refine the treatment options for these patients.

## Figures and Tables

**Table 1:** Current MODY subtypes—gene function, clinical manifestation and complications.

Gene Symbol	Full Name	Gene Function	Clinical Manifestation	Diabetic Complications
HNF1A	Hepatocyte nuclear factor 1 alpha	transcription factor	progressive insulin secretory defect; diminished renal threshold for glycosuria;	common
GCK	Glucokinase	enzyme in the first step of glucose metabolism	stable, mild fasting hyperglycemia;	rare
HNF4A	Hepatocyte nuclear factor 4 alpha	transcription factor	transient neonatal diabetes; progressive insulin secretory defect	common
HNF1B	Hepatocyte nuclear factor 1 beta	transcription factor	renal abnormalities and insufficiency at young age; liver test abnormalities; exocrine pancreatic dysfunction; hyperuricemia	common
PDX1	Pancreatic and duodenal homebox-1	transcription factor	permanent neonatal diabetes in homozygote; pancreas agenesis	unknown
NEUROD1	Neurogenic differentiation 1	transcription factor	neonatal diabetes; pancreatic abnormalities; child or adult-onset diabetes neurological abnormalities	unknown
KLF11	Krupell-like factor 11	transcription factor	pancreatic malignancy; similar to T2DM	unknown
CEL	Carboxyl ester lipase	controls exocrine and endocrine functions of pancreas	exocrine pancreatic dysfunction; lipomatosis and fibrosis with posterior diabetes development	unknown
PAX4	Paired box 4	transcription factor	possible ketoacidosis	unknown
INS	Insulin	encode the insulin precursor	permanent neonatal diabetes	unknown
BLK	B-lymphoid tyrosine kinase	tyrosine kinase functions in signal transduction	overweight	unknown
ABCC8	ATP-binding cassette C8	regulating insulin release	permanent or transient neonatal diabetes	unknown
KCNJ11	Inwardly rectifying potassium channel subfamily J member 11	regulating insulin release	neonatal diabetes in homozygote	unknown
APPL1	Adaptor protein, phosphotyrosine interacting with PH domain and leucine zipper 1	insulin signaling pathway	insulin secretion defect; child or adult-onset diabetes	unknown

**Table 2:** Summary of studies using pluripotent stem cells as disease modelling tool, hiPSCs—human induced pluripotent stem cells, ESCs—embryonic stem cells, ER—endoplasmic reticulum.

MODY Subtype	Pluripotent Cells	Differentiated Cell Type	Control Lines	Mechanism Revealed	Reference
HNF1B-MODY	Patient-derived hiPSCs	Pancreatic progenitors	Family non-diseased and non-family control individuals	Compensatory increase in PDX1 in mutant pancreatic progenitors.	[157]
HNF4A-MODY	Patient-derived hiPSCs	Insulin-producing beta-cells	Family non-diseased and non-family control individuals	No effect on expression of insulin genes, nor in the development of insulin-producing beta cells	[158]
HNF4A-MODY	Patient-derived hiPSCs	Hepatopancreatic progenitors (HPPs)	Family non-diseased and non-family control individuals	Alterations in hepatic and pancreatic beta-cell signatures and abnormal cytoplasmic localisation of HNF4A.	[156]
HNF1A-MODY	ESCs	Pancreatic beta-like cells	Isogenic control	Increase in alpha-cell gene expression markers, impaired insulin secretion, defect in glycolysis and mitochondrial respiration.	[159]
KCNJ11-MODY	ESCs (biallelic mutation introduced)	Pancreatic beta-like cells	Isogenic control	Impaired insulin secretion, defective glucose homeostasis	[160]
INS-MODY	Patient-derived hiPSCs	Pancreatic beta-like cells	Isogenic control	Increased expression of ER-stress associated genes, reduced proliferation in vitro, lower insulin secretion in vivo together with increased ER-stress markers.	[152]
HNF1A-MODY	hiPSCs	Endothelial cells	Isogenic control	Increased vascular permeability in response to pro-inflammatory cytokine, no difference in pro-angiogenic response.	[63]

## Supplementary Materials

The following are available online at https://www.mdpi.com/article/10.3390/ijms22147553/s1.
